# Pyrogen in Typhoid

**Published:** 1898-02

**Authors:** F. E. Watts

**Affiliations:** Olean, N. Y.


### PYROGEN IN TYPHOID.

F. E. Watts, M. D., Olean, N. Y.

Reading the case of typhoid and Hydrocyanic-acid in The Homœopathic Physician for November, 1897, page 449, reminded me of a case of typhoid fever I had last summer, and of the action of Pyrogen upon its progress.

A young lady of about twenty-four followed in the same school two other young ladies who had quit teaching on account of the fever, one going through a full course of typhoid. I was called to see her July 1st last. On the second day she became wild and delirious, presenting all the symptoms of typhoid fever.

On July 3d her morning temperature was 104, and evening 104 2-5, when I thought of what Dr. Swan had said of the action of Pyrogen, "It always abated the severity, often shortening the length of the fever, and never having any relapses." I gave a dose of Pyrogen, DMM., when that night she was less delirious, she being better the next day, and continued to do nicely under placebo until July 8th, when she had sinking spells, and they were afraid she would die. I gave another dose of Pyrogen, DMM. (Swan), although there was not an elevation of temperature. She improved at once under its action, and July 26th I discharged the case.

She commenced teaching school in a country district in September, and has continued her work in good health except to her chagrin in the fall her hair came out, so she had to cut it off short. Here is a record of the temperature:

| | | | | | |
|---|---|---|---|---|---|
| July 1, | 5.30 P. M., | 103 3-5° | July 8, | 10.00 A. M., | 102° |
| " 2, | 9.30 A. M., | 103 1-5° | " 8, | 5.10 P. M., Pyrogen, | 102° |
| " 2, | 5.30 P. M., | 104 2-5° | " 9, | 10.10 A. M., | 101 2-5° |
| " 3, | 9.30 A. M., | 104° | " 9, | 5.10 P. M., | 102 2-5° |
| " 3, | 5.30 P. M., Pyrogen, | 104 2-5° | " 10, | 9.30 A. M., | 98 1-2° |
| " 4, | 10.00 A. M., | 103 4-5° | " 10, | 6.00 P. M., | 101 2-5° |
| " 4, | 5.30 P. M., | 104° | " 11, | 10.30 A. M., | 100 2-5° |
| " 5, | 10.00 A. M., | 103 3-5° | " 11, | 6.40 P. M., | 100 3-5° |
| " 5, | 5.30 P. M., | 103 4-5° | " 12, | 10.20 A. M., | 98 1-2° |
| " 6, | 9.30 A. M., | 103 1-5° | " 12, | 5.30 P. M., | 100 3-5° |
| " 6, | 6.00 P. M., | 103 1-5° | " 13, | 10.00 A. M., | 98 2-5° |
| " 7, | 9.30 A. M., | 102 4-5° | " 13, | 5.30 P. M., | 99° |
| " 7, | 5.15 P. M., | 103 2-5° | | | |

From this time on the temperature was about normal, except July 25th, when it went to 100 degrees F.

The more I use the nosodes the better satisfied I am with their action.

